# Pathogen-Specific Alterations in the Gut Microbiota Predict Outcomes in Flare of Inflammatory Bowel Disease Complicated by Gastrointestinal Infection

**DOI:** 10.14309/ctg.0000000000000550

**Published:** 2022-12-08

**Authors:** Jordan E. Axelrad, Ze Chen, Joseph Devlin, Kelly V. Ruggles, Ken Cadwell

**Affiliations:** 1 Division of Gastroenterology, Department of Medicine, NYU Grossman School of Medicine, New York, New York, USA;; 2 Department of Microbiology, NYU Grossman School of Medicine, New York, New York, USA;; 3 Division of Precision Medicine, Department of Medicine, NYU Grossman School of Medicine, New York, New York, USA;; 4 Institute for Systems Genetics, NYU Grossman School of Medicine, New York, New York, USA.

**Keywords:** inflammatory bowel disease, flare, gastrointestinal infection, gastroenteritis

## Abstract

Enteric infection with *Clostridioides difficile*, *Escherichia coli* subtypes, and norovirus is commonly detected in flares of inflammatory bowel disease (IBD). We associated the gut microbiome during flare complicated by a gastrointestinal pathogen with outcomes of IBD.

**METHODS:**

We performed a cross-sectional study of 260 patients (92 IBD and 168 non-IBD) with a gastrointestinal polymerase chain reaction panel positive for *C. difficile, E. coli*, or norovirus, or negative during an episode of diarrhea from 2018 to 2020, and 25 healthy controls. Clinical variables, IBD status, and 2-year outcomes were collected. Using 16S rRNA sequencing, we measured the effect size of the gut microbiome on IBD characteristics and outcomes.

**RESULTS:**

There were major differences in the gut microbiome between patients with and without a pathogen and IBD. In IBD, a higher proportion of patients without a pathogen required hospitalization and IBD therapies at flare and within the 2 years after flare, driven by a milder disease course in flares complicated by an *E. coli* subtype or norovirus. Examining the contribution of clinical covariates, the presence of IBD, and C-reactive protein, *C. difficile* had a greater relative influence on the gut microbiome compared with the presence of an *E. coli* subtype or norovirus. In patients with *C. difficile* or no pathogen, lower microbiome diversity at flare was associated with adverse IBD outcomes over 2 years.

**DISCUSSION:**

Distinctive pathogen-specific gut microbiomes were associated with subsequent IBD outcomes. These findings may have direct implications for the management of IBD flares complicated by enteric pathogens.

## INTRODUCTION

Inflammatory bowel diseases (IBDs), comprising Crohn's disease (CD) and ulcerative colitis (UC), develop from a combination of genetic susceptibility and environmental factors that elicit a deleterious inflammatory response (1). The gut microbiota regulates mucosal immunity through a number of homeostatic pathways, and any disturbance to this dynamic equilibrium may result in disease (2,3). Gastrointestinal (GI) microbial dysbiosis and the subsequent immune response represents the critical relationship between environment and genetics that results in the cellular and phenotypic range of IBD (2–9). Cross-sectional studies have revealed unique gut microbiome signatures and immune cell populations for IBD subtypes, including UC, colonic CD, and ileal CD (5,10–14).

GI infection is a common cause of gut microbial dysbiosis, and several studies have demonstrated a link between enteric infection and IBD (6,15–17). Highly sensitive and specific stool molecular multiplex polymerase chain reaction (PCR)-based assays offer a rapid and accurate means of clinically diagnosing enteric infection (18–21). Using this technology, we have implicated enteric infection in nearly 30% of IBD flares, with the most common pathogens comprising *Clostridioides difficile, Escherichia coli,* and norovirus (16,17,21–24). We have also confirmed significant differences in the distribution of pathogens between symptomatic patients with CD or UC and without IBD (16).

Despite these observations and diagnostic advances, the relationship between enteric pathogens, the gut microbiome, and mechanisms that result in inflammation remains poorly understood. In addition, as broad stool molecular multiplex PCR testing does not differentiate colonization from infection, questions remain about the optimal interpretation of a positive result. Moreover, the similar presentations of enteric infection and flare of IBD present a significant clinical challenge. As such, the impact of enteric pathogens on the gut microbiome may help differentiate patients with an acute GI infection, flare of IBD, and concomitant flare of IBD complicated by an enteric pathogen. Moreover, the impact of enteric pathogens during and after flares remains unknown.

In this exploratory translational study, we aimed to characterize the gut microbiome during an acute enteric infection with *C. difficile, E. coli* subtypes, or norovirus in patients with and without IBD. We also aimed to evaluate the clinical presentation and management of patients with *C. difficile, E, coli* subtypes, or norovirus in patients with and without IBD.

## METHODS

### Study population

We performed a cross-sectional study of all inpatients and outpatients at New York University (NYU) Langone Health's Tisch Hospital, a quaternary care institution in New York City, who underwent stool testing with a FilmArray GI pathogen PCR panel (BioFire Diagnostics, Salt Lake City, UT) during an episode of acute GI symptoms from September 2018 to February 2019. GI symptoms included diarrhea, bloody diarrhea, hematochezia, abdominal pain, fever, nausea, and/or vomiting. We collected remnant stool specimens submitted to the microbiology laboratory at NYU Langone Health's Tisch Hospital after GI pathogen PCR panel clinical testing twice weekly. We stored 1 sample per patient. We also included a sample of 25 healthy controls without acute or chronic GI symptoms.

We specifically identified adult patient older than 18 years with a GI pathogen PCR panel positive for *C. difficile, E. coli* subtypes, or norovirus, or negative for all pathogens. We selected these pathogens because they represent approximately 90% of GI infections detected by pathogen diagnostics based on our previous data (17). Using the electronic medical record, we then identified patients with and without IBD. Separate testing for *C. difficile* was performed on all patients as part of their clinical care (Xpert C. difficile; Cepheid, Sunnyvale, CA), and all positives were detected by both modalities. Patients with multiple classes of pathogens identified in a single test were excluded. Patients with any chronic GI illness other than IBD (e.g., microscopic colitis, celiac disease, gut-vs-host disease, and irritable bowel syndrome) were excluded.

### Enteric pathogen testing

The GI pathogen panel PCR tests for 22 analytes in stool including 13 bacteria, 5 viruses, and 4 parasites including *Campylobacter* (*jejuni*, *coli*, and *upsaliensis*), *C. difficile* (Toxin A/B), *Plesiomonas shigelloides*, *Salmonella*, *Yersinia enterocolitica*, *Vibrio* (*parahaemolyticus*, *vulnificus*, and *cholerae*), *E. coli* subtypes (including enteroaggregative *E. coli*, enteropathogenic *E. coli*, enterotoxigenic *E. coli*, Shiga-like toxin-producing *E. coli*, *E. coli* O 157, and *Shigella*/enteroinvasive *E. coli*), *Cryptosporidium* species, *Cyclospora cayetanensis*, *Entamoeba histolytica*, *Giardia lamblia*, adenovirus F40/41, astrovirus, norovirus GI/GII, rotavirus A, and sapovirus (I, II, IV, and V). The GI pathogen panel PCR is capable of the simultaneous detection and identification of nucleic acids from multiple bacteria, viruses, and parasites directly from stool samples in Cary-Blair transport media. The multiplex PCR process takes approximately 1 hour. The clinical sensitivity and specificity is 94.5%–100% for all targets (18,25).

### Clinical variables

For clinical data, we recorded the following values from the medical record: presenting clinical symptoms, medication use, date of PCR test, PCR results, date of birth, place of PCR test (e.g., emergency department, outpatient visit, and inpatient hospitalization), sex, race, ethnicity, presence of IBD, date of IBD diagnosis, IBD subtype, IBD therapy use, inflammatory biomarkers (including C-reactive protein and erythrocyte sedimentation rate), and empiric and directed antimicrobial therapies for PCR results. Management and outcomes after testing were also recorded including hospitalization requirement, short-term and long-term management, and outcomes and utilization including IBD medications held, added, or up titrated; requirement for steroids; colectomy; other surgery; and subsequent endoscopy, radiology, hospitalization, emergency department visit, and IBD extension or complication. The severity of events was categorized from least to most severe as (1) no medical events (2), medications added or up titrated (3), hospitalization or emergency department visit, and (4) IBD-related surgery. Short-term events were classified as those occurring within 30 days, medium term within 1 year, and long term after 1 year.

### Fecal microbiome analysis

Genomic DNA was extracted following the recommendations of the International Human Microbiome Standards (26). For profiling microbiome composition, the hypervariable region (V4) of the bacterial and archaeal 16S rRNA gene was amplified by PCR using standardized techniques (27). For microbiome analysis, quality control was performed by using FastQC (28). We analyzed raw sequences through the Quantitative Insights Into Microbial Ecology 2 pipeline (29). Divisive Amplicon Denoising Algorithm 2-filtered reads were used for constructing amplicon sequence variants, followed by operational taxonomic unit classification using the Silva 138 99% (30). A description of the microbial community was derived from alpha (Chao1 and Shannon) and beta (weighted and unweighted UniFrac and Bray-Curtis) diversity estimates (31,32). Differential abundance analysis was performed by using linear discriminant analysis (LDA) effect size (Lefse) analysis (33).

### Outcomes and statistical analyses

Our primary outcome was specific microbiome signature using 16S bacterial rRNA gene sequencing associated with each pathogen and clinical scenario, including *C. difficile, E. coli,* norovirus, or negative for all pathogens, stratified by IBD status. To compare clinical variables among cohorts, Student t-test, Wilcoxon signed rank test, and χ^2^ analyses were used. For microbiome data, to calculate between-sample diversity, weighted and unweighted UniFrac metrics were applied to build phylogenetic distance matrices and used to construct hierarchical cluster trees using unweighted pair group method with arithmetic mean and principal coordinate analysis (PCoA) representations. The effects of clinical variables on the gut microbiome were analyzed by using the pseudo R square statistics values from R package multivariate distance matrix regression (34). The data visualization was performed by using R package phyloseq (35) and ggplot2 (36).

This protocol was approved by the Institutional Review Board at NYU Langone Health.

## RESULTS

### Study population and clinical variables

We identified 260 patients who underwent stool testing with a FilmArray GI pathogen PCR panel during an episode of acute GI symptoms from September 2018 to February 2019. Specifically, we targeted patients who tested positive for *C. difficile* (n = 53), *E. coli* subtypes (n = 55), or norovirus (n = 59), or negative for all pathogens on the panel (n = 93; Table 1). The sample of 25 healthy controls tested negative for all pathogens on the panel. Healthy controls comprised graduate student and postdoctoral laboratory members who consented to having a PCR panel performed on their stool sample.

**Table 1.** Baseline characteristics of patients who underwent stool testing with a FilmArray gastrointestinal pathogen PCR panel during an episode of acute gastrointestinal symptoms from September 2018 to February 2019

	Total, n = 260	Negative GI panel, n = 93	*Clostridioides difficile*, n = 53	*Escherichia coli* subtype, n = 55	Norovirus, n = 59	*P* value
Female	138 (53.1%)	52 (55.9%)	27 (50.9%)	34 (61.8%)	25 (42.4%)	0.187
Race						0.138
White	180 (69.2%)	73 (78.5%)	31 (58.5%)	38 (69.1%)	38 (64.4%)	
Black	24 (9.2%)	6 (6.5%)	9 (17%)	4 (7.3%)	5 (8.5%)	
Asian	12 (4.6%)	1 (1.1%)	5 (9.4%)	3 (5.5%)	3 (5.1%)	
Other/mixed	44 (16.9%)	13 (14%)	8 (15.1%)	10 (18.2%)	13 (22%)	
Hispanic ethnicity	39 (15%)	14 (15.1%)	9 (17%)	6 (10.9%)	10 (16.9%)	0.786
Age at test (yr; median, IQR)	43.4 (28.4–64.4)	42.8 (29.1–60.5)	49 (28.5–70.2)	40.5 (31.3–64.7)	42.4 (29–62.1)	
Place of test						0.001
Emergency department	70 (26.9%)	15 (16.1%)	16 (30.2%)	11 (20%)	28 (47.5%)	
Inpatient	107 (41.2%)	45 (48.4%)	25 (47.2%)	15 (27.3%)	22 (37.3%)	
Outpatient	82 (31.5%)	33 (35.5%)	12 (22.6%)	28 (50.9%)	9 (15.3%)	
Other	1 (0.4%)	0	0	1 (1.8%)	0	
Symptoms						
Diarrhea	179 (68.8%)	39 (41.9%)	40 (75.5%)	45 (81.8%)	55 (93.2%)	0.001
Bloody diarrhea	37 (14.2%)	20 (21.5%)	9 (17%)	7 (12.7%)	1 (1.7%)	0.007
Hematochezia	14 (5.4%)	6 (6.5%)	3 (5.7%)	4 (7.3%)	1 (1.7%)	0.536
Abdominal pain	143 (55%)	54 (58.1%)	28 (52.8%)	31 (56.1%)	30 (50.8%)	0.824
Fever	60 (23.1%)	18 (19.4%)	12 (22.6%)	13 (23.6%)	17 (28.8%)	0.607
Nausea/vomiting	106 (40.8%)	26 (28%)	16 (30.2%)	19 (34.5%)	45 (76.3%)	0.001
Other/unknown	26 (10%)	14 (15.1%)	6 (11.3%)	4 (7.3%)	2 (3.4%)	0.109
IBD	92 (35.4%)	54 (58.1%)	14 (26.4%)	15 (27.3%)	9 (15.3%)	0.001
Crohn's disease	41 (15.8%)	29 (31.2%)	4 (7.5%)	5 (9.1%)	3 (5.1%)	
Ulcerative colitis	51 (19.6%)	25 (26.9%)	10 (18.9%)	10 (18.2%)	6 (10.2%)	
Charlson Comorbidity Index	1 (0–3)	0 (0–1)	2 (0–5)	0 (0–3)	1 (0–4)	
Required hospitalization	103 (39.6%)	42 (45.2%)	24 (45.3%)	15 (27.3%)	22 (37.3%)	0.136
Length of stay (median, IQR)	5 (3–9)	6 (3–9)	6 (3–11)	6 (4–9)	3 (2–7)	
Antimicrobial requirement	130 (50%)	27 (29%)	53 (100%)	40 (72.7%)	22 (37.3%)	0.001
As empirical therapy	71 (54.6%)	27 (29%)	21 (39.6%)	15 (37.5%)	22 (37.3%)	0.005
Empirical therapy discontinued	20/71 (28.2%)	1/27 (3.7%)	8/21 (38.1%)	5/15 (33.3%)	6/22 (27.7%)	0.025
Directed therapy for specific pathogen	75 (72.8%)	0	35 (66%)	32 (58.2%)	0	0.001
Healthcare utilization after test						
Endoscopy	37 (15%)	21 (24.7%)	8 (16%)	6 (11.1%)	2 (3.5%)	0.005
Radiology	96 (38.9%)	47 (55.3%)	27 (52.9%)	11 (20.4%)	11 (19.3%)	0.001

GI, gastrointestinal; IBD, inflammatory bowel disease; IQR, interquartile range.

There were no major demographic or clinical differences between patients who tested negative or positive for a pathogen (Table 1). Compared with patients with an *E. coli* subtype or norovirus, those with *C. difficile* tended to be older, have a higher median Charlson Comorbidity Index, were more frequently tested in the inpatient setting, and more likely to require hospitalization, antimicrobial therapy, and radiography after stool testing. Stratifying by IBD, we identified 92 patients (35.4% of the total population, 41 with CD and 51 with UC) who tested positive for *C. difficile* (n = 14, 15.2%), *E. coli* subtypes (n = 15, 16.3%), or norovirus (n = 9, 9.8%), or negative for all pathogens on the panel (n = 54, 58.7%; Table 2). Most patients with IBD were on an IBD therapy at the time of stool testing and had a disease duration greater than 10 years. There were no major differences in demographic or clinical differences between patients with IBD with or without a pathogen; however, patients with IBD with a pathogen tended to present with more diarrhea and nausea or vomiting and had lower biomarkers of IBD activity at stool testing (see Supplementary Table 1, http://links.lww.com/CTG/A896).

**Table 2.** Baseline characteristics of patients who underwent stool testing stratified by IBD status

	Total IBD, n = 92	Crohn's disease, n = 41	Ulcerative colitis, n = 51
Pathogen			
No pathogen	54 (58.7%)	29 (70.7%)	25 (49%)
*Clostridioides difficile*	14 (15.2%)	4 (9.8%)	10 (19.6%)
*Escherichia coli* subtype	15 (16.3%)	5 (12.2%)	10 (19.6%)
Norovirus	9 (9.8%)	3 (7.3%)	6 (11.8%)
Female	46 (50%)	18 (43.9%)	28 (54.9%)
Age at test (yr; median, IQR)	35.5 (27.2–53.5)	42.3 (26.4–54.9)	33.2 (28.2–46.4)
Montreal classification CD			
A1–age <17	14 (15.2%)	14 (34.1%)	—
A2–age 17-40	13 (14.1%)	18 (43.9%)	—
A3–age >40	9 (9.8%)	9 (22%)	—
L1–ileal	9 (9.8%)	9 (22%)	—
L2–colonic	8 (8.7%)	8 (19.5%)	—
L3–ileocolonic	24 (26.1%)	24 (58.5%)	—
B1–inflammatory	14 (15.2%)	14 (34.1%)	—
B2–stricturing	3 (3.3%)	3 (7.3%)	—
B3–penetrating	24 (26.1%)	24 (58.5%)	—
Perianal	9 (9.8%)	9 (22%)	—
Montreal classification UC			
E1–proctitis	10 (10.9%)	—	10 (19.6%)
E2–left-sided colitis	12 (13%)	—	12 (23.5%)
E3–extensive colitis	29 (31.5%)	—	29 (56.8%)
Duration of IBD (yr; median, IQR)	10.1 (2.2–143)	12.4 (5.3–18.3)	3.3 (0.9–12.8)
IBD therapies at test			
Systemic steroids	16 (17.4%)	4 (9.8%)	12 (23.5%)
Topical steroids	9 (9.8%)	3 (7.3%)	6 (11.8%)
5-ASA	29 (31.5%)	10 (24.4%)	19 (37.3%)
Anti-TNF	15 (16.3%)	8 (19.5%)	7 (13.7%)
Anti-integrin	6 (6.5%)	1 (2.4%)	5 (9.8%)
Anti-IL 12/23	6 (6.5%)	6 (14.6%)	0
JAK inhibitor	1 (1.1%)	0	1 (2%)
Immunomodulator	6 (6.5%)	4 (9.8%)	2 (3.9%)
None	25 (27.2%)	12 (29.3%)	13 (25.5%)
Place of test			
Emergency department	17 (18.5%)	11 (26.8%)	6 (11.8%)
Inpatient	47 (51.1%)	23 (56.1%)	24 (47.1%)
Outpatient	28 (30.4%)	7 (17.1%)	21 (41.2%)
Symptoms			
Diarrhea	38 (41.3%)	17 (41.5%)	21 (41.2%)
Bloody diarrhea	28 (30.4%)	4 (9.8%)	24 (47.1%)
Hematochezia	5 (5.4%)	0	5 (9.8%)
Abdominal pain	54 (58.7%)	24 (58.5%)	30 (58.8%)
Fever	18 (19.6%)	11 (26.8%)	7 (13.7%)
Nausea/vomiting	28 (30.4%)	14 (34.1%)	14 (27.5%)
Other/unknown	12 (13%)	6 (14.6%)	6 (11.8%)
Inflammatory biomarkers (median, IQR)			
C-rp (mg/L)	15.9 (4.2–82)	34 (4.8–108)	10.1 (2.6–53.1)
ESR (mm/hr)	28 (9–80)	54 (20–85)	26 (9–61)

5-ASA, aminosalicylates; CD, Crohn's disease; C-rp, C-reactive protein; ESR, sedimentation rate; IBD, inflammatory bowel disease; IL, interleukin; IQR, interquartile range; JAK, Janus kinase; TNF, tumor necrosis factor; UC, ulcerative colitis.

### Microbiome analyses

**Figure 1.**
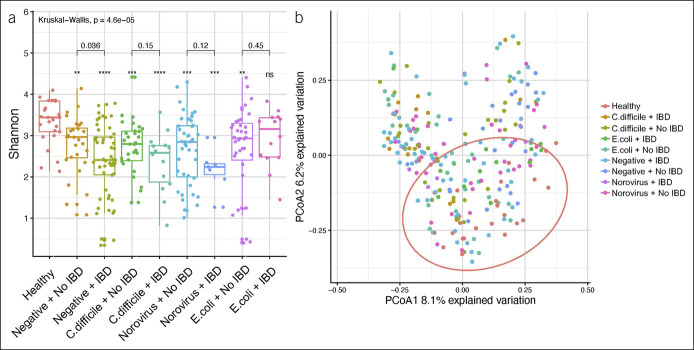
Microbiome diversity by (**a**) Shannon index between patients with and without a pathogen and (**b**) principal coordinates analysis (PCoA). *Clostridioides difficile, Escherichia coli*, inflammatory bowel disease (IBD).

There were no major differences in microbiome diversity by Shannon index (H) between patients with and without a pathogen (Figure 1a). However, in patients negative for all pathogens, diversity was reduced in patients with IBD compared with patients without IBD. Diversity was substantially greater in healthy controls compared with all other patient groups. These findings were further investigated by PCoA, demonstrating moderate separation of healthy controls from other IBD and pathogen cohorts (Figure 1b).

**Figure 2.**
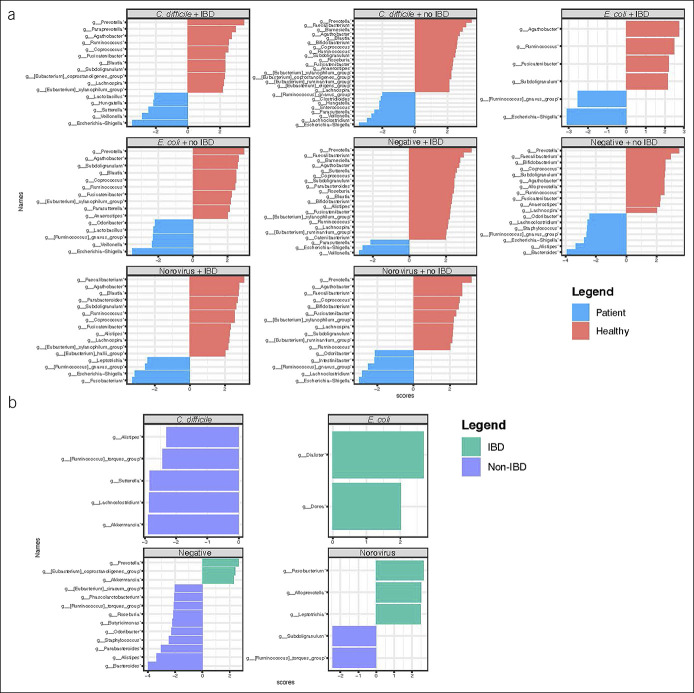
(**a**) LEfSe plots normalized to healthy controls by each pathogen and inflammatory bowel disease (IBD) status and (**b**) within patients with IBD by pathogen. *Clostridioides difficile, Escherichia coli*, inflammatory bowel disease (IBD).

There were major differences in gut microbiota community structure between patients with and without the presence of a GI pathogen. Stratifying by IBD and pathogen on a clustered heatmap normalized to healthy controls, there were several differentially abundant microbes denoting a specific gut microbial signature associated with each pathogen and by the presence of IBD (see Supplementary Figure http://links.lww.com/CTG/A906). According to LEfSe analysis, patients with *C. difficile* were enriched in *Lachnoclostridium*, *Veillonella,* and *Escherichia*; patients with *E. coli* subtypes were enriched in *Ruminococcus gnavus and Escherichia*; and patients with norovirus were enriched in *Ruminococcus gnavus, Fusobacterium, and Escherichia* (Figure 2a). On differential abundance analysis between patients with and without IBD, *Akkermansia and Lachnoclostridium* were increased in *C. difficile* without IBD; *Dorea* and *Dialister* were increased in *E.coli* with IBD; *Alloprevotella* and *Fusobaterium* were increased in norovirus with IBD; and *Roseburia, Alistipes,* and *Bacteroides* were increased in those without IBD or a pathogen (Figure 2b).

**Figure 3.**
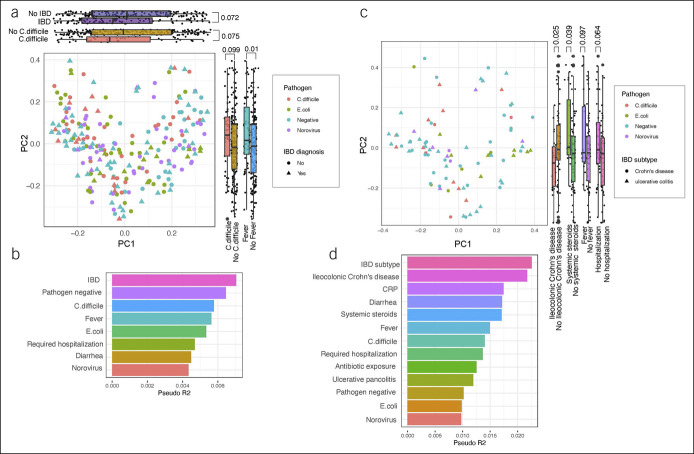
(**a**) Bray-Curtis dissimilarity in patients with *Clostridioides difficile,* an *Escherichia coli* subtype, or norovirus, or negative for any pathogen with the (**b**) contribution of clinical covariates at the time of stool testing to Bray-Curtis dissimilarity-based principal coordinates analysis (PCoA). (**c**) Bray-Curtis dissimilarity in patients with inflammatory bowel disease (IBD) with *C. difficile*, an *E. coli* subtype, or norovirus, or negative for any pathogen with the (**d**) contribution of clinical covariates at the time of stool testing to Bray-Curtis dissimilarity-based PCoA analysis. C-reactive protein (CRP).

On Bray-Curtis dissimilarity, patients with *C. difficile,* an *E. coli* subtype, or norovirus differed from patients negative for any pathogen (Figure 3a). Examining the contribution of various clinical covariates at the time of stool testing to Bray-Curtis dissimilarity-based PCoA analysis, the presence of IBD and a pathogen had the largest relative influence on the gut microbiome compared with other covariates, including symptoms or hospitalization requirement (Figure 3b). However, individual pathogens, specifically an *E. coli* subtype or norovirus, had a relatively smaller relative influence on the gut microbiome compared with the absence of a pathogen or *C. difficile*. Focusing on patients with IBD only, the presence of *C. difficile,* an *E. coli* subtype, or norovirus differed from patients negative for any pathogen (Figure 3c). However, in patients with IBD, examining the contribution of various clinical covariates at the time of stool testing to Bray-Curtis dissimilarity-based PCoA analysis, IBD subtypes and biomarkers of inflammation such as C-reactive protein had larger relative influences on the gut microbiome compared with the presence of *an E. coli* subtype or norovirus (Figure 3d).

### Management and outcomes

**Figure 4.**
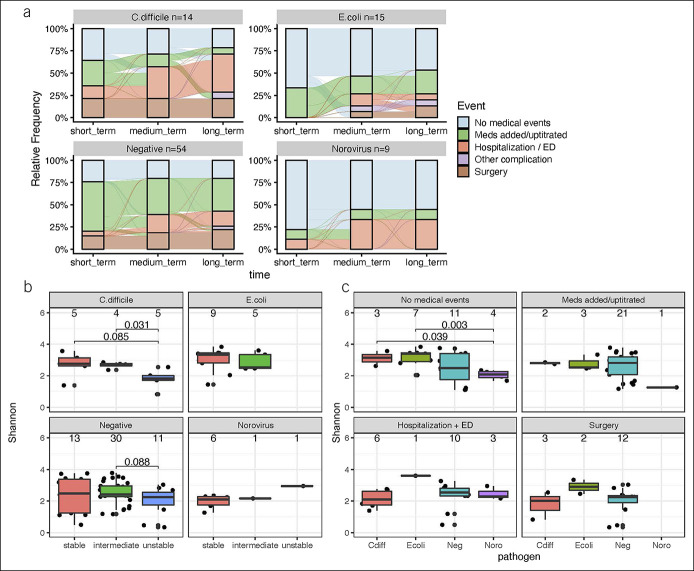
(**a**) Flowchart of adverse outcomes and utilization requirements in patients with inflammatory bowel disease (IBD) and *Clostridioides difficile,* an *Escherichia coli* subtype, or norovirus over time after stool testing. Samples are shown as a percent of that total samples in that category. (**b**) Shannon diversity by number of subsequent adverse outcomes and utilization requirements (none = stable, 1 = intermediate, and >1 = unstable). (**c**) Diversity by Shannon index and future adverse outcomes and utilization requirements. Emergency department (ED).

For IBD management, a greater proportion of patients with IBD but without a pathogen required hospitalization compared with those with a pathogen (59.3% vs 39.5%), largely driven by a lower hospitalization rate in those with an *E. coli* subtype (26.7%) or norovirus (33.3%; Table 3). Accordingly, a greater proportion of patients with IBD without a pathogen required additional or escalated IBD therapy, including corticosteroids and surgery, mainly driven by lower requirements for these management approaches in those with an *E. coli* subtype or norovirus. Within 30 days and 2 years after stool testing, a greater proportion of patients with IBD but without a pathogen used health care or experienced an adverse IBD outcome compared with those with a pathogen (64.8% and 66.7% vs 47.4% and 57.9%, respectively). Focusing on specific pathogens, patients with IBD and *C. difficile* experienced similar outcomes and utilization requirements to those without a pathogen (Figure 4a). Patients with IBD and an *E. coli* subtype or norovirus experienced similar outcomes and utilization requirements.

**Table 3.** IBD management of flares with and without a gastrointestinal pathogen and subsequent IBD outcomes

	IBD and negative GI panel, n = 54	IBD and any pathogen, n = 38	*P* value	*Clostridioides difficile*, n = 14	*Escherichia coli* subtype, n = 15	Norovirus, n = 9
Required hospitalization	32 (59.3%)	15 (39.5%)	0.062	8 (57.1%)	4 (26.7%)	3 (33.3%)
Length of stay (median, IQR)	6 (4–10)	4 (2–7)		6 (3–7)	4 (3–6)	2 (2–8)
Antimicrobial requirement	15 (28.3%)	27 (71.1%)		14 (100%)	11 (78.6%)	2 (22.2%)
As empirical therapy	11 (73.3%)	7 (25.9%)	0.001	4 (28.6%)	1 (9.1%)	2 (100%)
Empirical therapy discontinued	0	3 (42.9%)	0.001	4 (100%)	0	1 (50%)
Directed therapy for pathogen	0	22/27 (81.5%)	0.017	12 (85.7%)	10 (90.9%)	0
IBD management						
No change	14 (25.9%)	21 (55.3%)	0.004	7 (50%)	7 (46.7%)	7 (77.8%)
Medications held	0	0	—	0	0	0
Medications added/up titrated	14 (25.9%)	7 (18.4%)	0.398	4 (28.6%)	2 (12.3%)	1 (11.1%)
Steroid requirement	29 (54.7%)	10 (26.3%)	0.315	6 (42.9%)	4 (26.7%)	0
Colectomy	3 (5.6%)	2 (5.3%)	0.951	2 (14.2%)	0	0
Other surgery	8 (15.1%)	3 (8.1%)	0.319	3 (21.4%)	0	0
30-d utilization						
Endoscopy	14 (26.4%)	10 (27%)	0.948	5 (35.7%)	5 (35.7%)	0
Radiology	32 (60.4%)	11 (28.7%)	0.004	8 (57.1%)	1 (7.1%)	2 (22.2%)
Hospitalization	2 (3.8%)	3 (7.9%)	0.395	3 (21.4%)	0	0
ED visit	3 (5.7%)	2 (5.3%)	0.935	1 (7.1%)	0	1 (11.1%)
Any 30-d utilization	35 (64.8%)	18 (47.4%)	0.134	9 (64.3%)	6 (40%)	3 (33.3%)
2-yr utilization and outcomes						
Disease extension	11 (20.4%)	8 (21.1%)	0.937	3 (21.4%)	2 (13.3%)	3 (33.3%)
IBD therapy added/up titrated	26 (48.1%)	10 (26.3%)	0.035	5 (36.7%)	5 (33.3%)	0
Steroid requirement	15 (27.8%)	9 (23.7%)	0.660	4 (28.6%)	2 (13.3%)	3 (33.3%)
Colectomy	2 (3.7%)	0	0.230	0	0	0
Other surgery	6 (11.1%)	4 (10.5%)	0.929	2 (14.3%)	2 (13.3%)	0
Hospitalization	17 (31.5%)	11 (28.9%)	0.795	6 (42.9%)	3 (20%)	2 (22.2%)
ED visit	7 (13%)	10 (26.3%)	0.104	5 (35.7%)	3 (20%)	2 (22.2%)
Other complication	5 (9.3%)	3 (7.9%)	0.819	1 (7.1%)	2 (13.3%)	0
Any 2-yr utilization or outcome	36 (66.7%)	22 (57.9%)	0.51	9 (64.3%)	8 (53.3%)	5 (55.6%)

IBD, inflammatory bowel disease; IQR, interquartile range; ED, emergency department.

We then went on to examine the relationship between the gut microbiome at the time of stool testing and subsequent IBD management and outcomes. Although somewhat limited by sample size and events for *E. coli* subtypes or norovirus, there were modest differences based on pathogen status with norovirus having a significantly lower diversity by the Shannon index compared with *E. coli* and *C. difficile* subtypes for those with no medical events (Figure 4c). Classifying patients by number of subsequent adverse outcomes (none = stable, 1 = intermediate, and >1 = unstable), lower diversity by the Shannon index was associated with an increasing number of future adverse outcomes (Figure 4b). Sample-level Shannon diversity values and statistical outputs can be found in the Supplementary Digital Content (see Supplementary Table 2, http://links.lww.com/CTG/A897).

## DISCUSSION

In this cross-sectional study of patients with *C. difficile*, an *E. coli* subtype, norovirus, or negative for pathogens during an episode of acute GI symptoms, gut microbiomes distinguish and differentially respond to pathogens in patients with and without IBD. Of all patients, those with *C. difficile* or negative for pathogens tended to represent a sicker cohort of patients compared with those with an *E. coli* subtype or norovirus, with higher rates of hospitalization, healthcare utilization, and in patients with IBD, worse IBD outcomes over 2 years of follow-up. These data suggest that in patients with IBD and an exacerbation in GI symptoms, the identification of an *E. coli* subtype or norovirus, the most common pathogens detected during flare after *C. difficile* (16,17), may represent a unique molecular phenotype of flare that is milder, whereas the presence of *C. difficile* may represent a unique, more severe subset of flare.

A defective intestinal barrier is a feature of both IBD and enteric infection. A recent study demonstrated that aberrant intestinal permeability precedes a diagnosis of CD, suggesting that pathogen-induced disruption of the intestinal barrier may be a contributing factor (37). Specific pathogens might cause gut microbial dysbiosis, worsen existing dysbiosis, alter immune responses, or activate immune responses through direct damage, all potentially triggering disease exacerbation as a unique pathogen-induced flare subset (38). The role of the gut microbiome in both IBD and enteric infection is evolving. In a study of diarrheagenic *E. coli*, a *Ruminiclostridium* species was associating with resistance to gastroenteritis and an uncultured Ruminococcaceae taxon was associated with susceptibility, in addition to an observation of a disrupted microbiota several weeks before diarrhea, which could serve as an indicator of susceptibility (39). In a recent study of children with IBD and *C. difficile* infection, IBD produced a gut microbial environment conducive to subsequent *C. difficile* infection and active *C. difficile* correlated with the production of distinctive fermentation products, including a novel metabolite, isocaproyltaurine, that linked taurine produced during inflammation with a distinctive *C. difficile* fermentation product, isocaproate (40). These findings confirmed unique microbial and metabolic processes and provide potential phenotype biomarkers of IBD complicated by *C. difficile*.

Although PCR testing fails to discriminate between active infection and colonization, and there is uncertainty regarding the clinical interpretation of GI pathogen multiplex assays (41), the probability of carriage in this symptomatic cohort was likely low. In fact, although representing a small sample, no healthy control in our study tested positive for any GI pathogen on the panel. In a recent study of 333 patients with IBD and 52 healthy controls, a significantly higher percentage of actively inflamed patients had positive stool tests (31%) compared with those with quiescent disease (8%, *P* < 0.001) and healthy controls (14%, *P* = 0.01) (42). Despite these previous data demonstrating low detection of pathogens in healthy controls and quiescent IBD, the possibility of reverse causality, active IBD increasing the risk of infection or colonization acquisition, and that these species are merely “innocent bystanders” remains unresolved and complicates our understanding of IBD (38,43).

Specifically, *C. difficile* carriage more often occurs in the setting of reduced gut microbiome diversity, as occurs after antibiotic exposure, with reduced diversity predisposing to *C. difficile* colonization with or without clinical infection (44). This observation further complicates our understanding of its role in IBD pathogenesis and contributes to the reverse causality consideration because reduced diversity might predispose to IBD and reduced diversity also occurs as a result of IBD. However, our previous studies have demonstrated a lower overall pathogen detection rate in patients with an exacerbation of existing IBD compared with non-IBD controls with acute GI symptoms (16), and although patients with IBD may have an impaired intestinal barrier with dysfunctional antibacterial activity, this deficit does not directly translate into an increased risk of GI pathogen acquisition or infection. Thus, the above clinical and microbial data, which distinguish based on the presence of IBD and pathogens, provide new insight and hypotheses for our understanding of factors influencing IBD.

There are several limitations to the current study inherent to a cross-sectional and retrospective study design. Our analyses do not prove a cause-and-effect relationship between GI symptoms, enteric infection, and the gut microbiota. Moreover, we could not retrospectively confirm the presence or absence of endoscopic disease activity in patients with IBD, precluding the ability to definitively establish a relationship between GI infection and flare of IBD. Thus, we could not differentiate between GI infection and flare complicated by GI infection. As such, patients with IBD may have experienced gastroenteritis without true flare or an appreciable impact on the disease course of IBD. Testing was performed in a nonrandom manner, and other factors may have influenced decision making in physician ordering. Individual patient information concerning precise presenting symptoms, medication exposures, other comorbid conditions, and management after stool testing was subject to documentation in the medical record, including inflammatory biomarkers. In addition, thresholds for testing in specific populations may influence rates of overall infection and the gut microbiota. Although the patient population was racially and ethnically diverse, most patients resided in the Northeast United States. The FilmArray GI pathogen PCR panel does not assess for the presence of cytomegalovirus, a pathogen of increasing importance in IBD.

In summary, we demonstrate that the gut microbiota differentially responds to the presence of *C. difficile*, *E. coli* subtypes, or norovirus in patients with GI symptoms. In patients with IBD, specific pathogens have a distinct phenotypic impact on the gut microbiome and on subsequent IBD outcomes, suggesting a role in disease pathogenesis beyond an “innocent bystander.” Ongoing prospective studies are required to clinically and molecularly differentiate enteric infection from flare complicated by a GI pathogen to identify optimal management strategies for this common clinical scenario.

## CONFLICTS OF INTEREST

**Guarantor of the article:** Jordan E. Axelrad, MD, MPH.

**Specific author contributions:** Study concept and design: all co-authors. Acquisition of data: J.E.A. Writing first draft of the manuscript: J.E.A. Critical revision of the manuscript for important intellectual content and approval of final version: all co-authors. All authors approved the final version of the article, including the authorship list.

**Financial support:** J.E.A. receives research support from the Crohn's and Colitis Foundation, the Judith and Stewart Colton Center for Autoimmunity, and the NIH NIDDK Diseases K23DK124570. K.C. has recently been supported by NIH grants DK093668, AI121244, HL123340, AI130945, AI140754, and DK124336; pilot awards from the NYU Cancer Center grant P30CA016087 and Judith and Stewart Colton Center of Autoimmunity; and Faculty Scholar grant from the Howard Hughes Medical Institute, Crohn's and Colitis Foundation, Kenneth Rainin Foundation, and PATH award from the Burroughs Wellcome Fund.

**Potential competing interests:** J.E.A. reports receiving research grants from BioFire Diagnostics; consultancy fees or honorarium from BioFire Diagnostics, AbbVie, Pfizer, BMS, and Janssen; and holds US patent 2012/0052124A1. K.C. reports receiving research funding from Pfizer, Takeda, and AbbVie; consultancy fees or honorarium from PureTech Health, Genentech, and AbbVie; and holds US patent 10,722,600 and provisional patent 62/935,035.

**IRB:** This protocol was approved by the Institutional Review Board at NYU Langone Health.

## Supplementary Material

SUPPLEMENTARY MATERIAL

## Notes

Study Highlights WHAT IS KNOWN

✓ *Clostridioides difficile, Escherichia coli* subtypes, and norovirus are commonly detected in flares of inflammatory bowel disease.
✓ The impact of enteric pathogens during and after flares remains unknown.

WHAT IS NEW HERE

✓ There were major differences in the gut microbiota between patients with and without an enteric pathogen and inflammatory bowel disease.
✓ Pathogens produced a specific gut microbiome signature during flare that was associated with subsequent outcomes.
