# Supplementary material for: Pathogen-Specific Alterations in the Gut Microbiota Predict Outcomes in Flare of Inflammatory Bowel Disease Complicated by Gastrointestinal Infection
Source: Clin Transl Gastroenterol. 2022 Dec 8;14(2):e00550. doi: 10.14309/ctg.0000000000000550 (PMC9945377; doi:10.14309/ctg.0000000000000550)
Supplement: SUPPLEMENTARY MATERIAL [file ct9-14-e00550-s001.docx]

| Supplemental Table 1 | IBD and negative GI panel  n= 54 | IBD and any pathogen  n= 38 | p-value | Clostridioides difficile  n= 14 | Escherichia coli subtype  n= 15 | Norovirus  n= 9 |
| --- | --- | --- | --- | --- | --- | --- |
| Female | 29 (53.7%) | 17 (44.7%) | 0.397 | 4 (28.6%) | 10 (66.7%) | 3 (33.3%) |
| Age at test (years; median, IQR) | 36 (27.8-55.4) | 34 (26.6-43.3) | 0.300 | 32.5 (26.6-46.4) | 33.2 (26.9-43.3) | 37.1 (24.4-42.3) |
| IBD  Crohn’s disease  Ulcerative colitis | 29 (53.7%)  25 (46.3%) | 12 (31.6%)  26 (68.4%) | 0.036 | 4 (28.6%)  10 (71.4%) | 5 (33.3%)  10 (66.7%) | 3 (33.3%)  6 (66.7%) |
| Montreal classification CD  A1- Age <17  A2- Age 17-40  A3- Age >40  L1- Ileal  L2- Colonic  L3- Ileocolonic  B1- Inflammatory  B2- Stricturing  B3- Penetrating  Perianal | 11 (20.4%)  12 (41.4%)  6 (11.1%)  6 (11.1%)  5 (9.3%)  18 (33.3%)  11 (20.4%)  1 (1.9%)  17 (31.5%)  7 (13%) | 3 (7.9%)  6 (15.8%)  3 (7.9%)  3 (7.9%)  3 (7.9%)  6 (15.8%)  2 (5.3%)  2 (5.3%)  8 (21.1%)  2 (5.3%) | 0.609  0.819  0.160  0.221 | 0  4 (28.6%)  0  1 (7.1%)  1 (7.1%)  2 (14.3%)  1 (7.1%)  0  3 (21.4%)  2 (14.3%) | 2 (13.3%)  1 (6.7%)  2 (13.3%)  2 (13.3%)  0  3 (20%)  1 (6.7%)  2 (13.3%)  2 (13.3%)  0 | 1 (11.1%)  1 (11.1%)  1 (11.1%)  0  2 (22.2%)  1 (11.1%)  0  0  3 (33.3%)  0 |
| Montreal classification UC  E1- Proctitis  E2- Left-sided colitis  E3- Extensive colitis | 5 (9.3%)  5 (9.3%)  15 (27.8%) | 5 (13.2%)  7 (18.4%)  14 (36.8%) | 0.693 | 2 (14.3%)  2 (14.3%)  6 (42.9%) | 3 (20%)  2 (13.3%)  5 (33.3%) | 0  3 (33.3%)  3 (33.3%) |
| Duration of IBD (years; median, IQR) | 7.9 (1.3-16.5) | 10.5 (2.5-13.9) | 0.621 | 8.4 (1.5-13.8) | 11.5 (3.3-14.3) | 10.5 (3.6- 13.3) |
| IBD therapies at test  Systemic steroids  Topical steroids  5-ASA  Anti-TNF  Anti-Integrin  Anti-IL 12/23  JAK inhibitor  Immunomodulator  None | 13 (24.1%)  4 (7.4%)  13 (24.1%)  10 (18.5%)  2 (3.7%)  6 (11.1%)  1 (1.9%)  3 (5.6%)  14 (25.9% | 3 (7.9%)  5 (13.2%)  16 (42.1%)  5 (13.2%)  4 (10.5%)  0  0  3 (7.9%)  11 (28.9%) | 0.044  0.361  0.067  0.493  0.192  0.034  0.399  0.655  0.748 | 2 (14.3%)  0  7 (50%)  2 (14.3%)  1 (7.1%)  0  0  1 (7.1%)  3 (21.4%) | 0  4 (26.7%)  6 (40%)  2 (13.3%)  3 (20%)  0  0  1 (6.7%)  4 (26.7%) | 1 (11.1%)  1 (11.1%)  3 (33.3%)  1 (11.1%)  0  0  0  1 (11.1%)  4 (44.4%) |
| Place of test  Emergency department  Inpatient  Outpatient | 9 (16.7%)  32 (59.3%)  13 (24.1%) | 8 (21.1%)  15 (39.5%)  15 (39.5%) | 0.159 | 1 (7.1%)  8 (57.1%)  5 (25.7%) | 2 (13.3%)  4 (26.7%)  9 (60%) | 5 (55.6%)  3 (33.3%)  1 (11.1%) |
| Symptoms  Diarrhea  Bloody diarrhea  Hematochezia  Abdominal pain  Fever  Nausea/vomiting  Other | 12 (22.2%)  19 (35.2%)  2 (3.7%)  31 (57.4%)  13 (24.1%)  12 (22.2%)  7 (13%) | 26 (68.4%)  9 (23.7%)  3 (7.9%)  23 (60.5%)  5 (13.2%)  16 (42.1%)  5 (13.2%) | 0.001  0.238  0.383  0.765  0.194  0.041  0.978 | 10 (71.1%)  5 (35.7%)  0  8 (57.1%)  1 (7.1%)  4 (28.6%)  2 (14.3%) | 9 (60%)  4 (26.7%)  3 (20%)  8 (53.3%)  2 (13.3%)  3 (20%)  3 (20%) | 7 (77.9%)  0  0  7 (77.8%)  2 (22.2%)  9 (100%)  0 |
| Inflammatory biomarkers (median, IQR)  C-rp (mg/L)  ESR (mm/hr) | 28 (5.4-94)  57 (23-85) | 11.3 (2-47.6)  12 (8-28) | 0.088  0.014 | 12.6 (1.4-74)  26 (12-86) | 6.7 (2.8-14.4)  8 (5-50) | 37.2 (21.4-57.9)  11 (9-12) |

Abbreviations: IBD- inflammatory bowel disease; IQR- interquartile range; 5-ASA- aminosalicylates; TNF- tumor necrosis factor; IL- interlukin; JAK- Janus kinase; C-rp- C-reactive protein; ESR- sedimentation rate.
