# Supplementary material for: Pathogen-Specific Alterations in the Gut Microbiota Predict Outcomes in Flare of Inflammatory Bowel Disease Complicated by Gastrointestinal Infection
Source: Clin Transl Gastroenterol. 2022 Dec 8;14(2):e00550. doi: 10.14309/ctg.0000000000000550 (PMC9945377; doi:10.14309/ctg.0000000000000550)
Supplement: SUPPLEMENTARY MATERIAL [file ct9-14-e00550-s002.docx]

**Supplementary Table 2.** (A) Shannon diversity index values for each sample. (B) Statistical output comparing adverse outcomes within IBD subtypes. (C) Statistical output comparing adverse outcomes within pathogen negative subtypes. (D) Statistical output comparing adverse outcomes within pathogen positive subtypes.

| A | long_term | pathogen | shannon |
| --- | --- | --- | --- |
| Cdiff05 | Hospitalization + ER | Cdiff | 1.7128828 |
| Cdiff17 | Hospitalization + ER | Cdiff | 1.393144 |
| Cdiff22 | Meds added/uptitrated | Cdiff | 2.74694162 |
| Cdiff28 | surgery | Cdiff | 0.8287393 |
| Cdiff30 | No medical events | Cdiff | 3.56665871 |
| Cdiff31 | Hospitalization + ER | Cdiff | 1.83054028 |
| Cdiff32 | Hospitalization + ER | Cdiff | 2.76347052 |
| Ecoli14 | Meds added/uptitrated | Ecoli | 3.34045912 |
| Ecoli26 | surgery | Ecoli | 2.46050394 |
| Ecoli28 | No medical events | Ecoli | 3.83331178 |
| Ecoli31 | surgery | Ecoli | 3.34315157 |
| Ecoli32 | No medical events | Ecoli | 2.80628062 |
| Neg10 | Meds added/uptitrated | Neg | 2.4273418 |
| Neg15 | No medical events | Neg | 3.43689551 |
| Neg20 | surgery | Neg | 3.0372995 |
| Neg27 | No medical events | Neg | 1.23604295 |
| Noro06 | Meds added/uptitrated | Noro | 1.26576771 |
| Noro17 | No medical events | Noro | 1.94690153 |
| Noro22 | Hospitalization + ER | Noro | 2.16723548 |
| Cdiff37 | surgery | Cdiff | 2.54619035 |
| Cdiff38 | Hospitalization + ER | Cdiff | 2.37181686 |
| Cdiff41 | No medical events | Cdiff | 2.62222384 |
| Cdiff43 | No medical events | Cdiff | 3.13526913 |
| Cdiff48 | Hospitalization + ER | Cdiff | 2.71511469 |
| Cdiff49 | surgery | Cdiff | 2.01479098 |
| Cdiff51 | Meds added/uptitrated | Cdiff | 2.84990628 |
| Ecoli35 | No medical events | Ecoli | 2.97899763 |
| Ecoli40 | No medical events | Ecoli | 3.44071664 |
| Ecoli41 | Meds added/uptitrated | Ecoli | 2.52853917 |
| Ecoli44 | Hospitalization + ER | Ecoli | 3.60122427 |
| Ecoli49 | No medical events | Ecoli | 2.04048034 |
| Ecoli52 | Meds added/uptitrated | Ecoli | 2.46917508 |
| Ecoli54 | No medical events | Ecoli | 3.40059428 |
| Ecoli55 | No medical events | Ecoli | 3.45283439 |
| Neg36 | surgery | Neg | 2.10042745 |
| Neg37 | Meds added/uptitrated | Neg | 1.54648482 |
| Neg38 | Meds added/uptitrated | Neg | 3.78181831 |
| Neg40 | Meds added/uptitrated | Neg | 1.47672851 |
| Neg41 | surgery | Neg | 0.34303583 |
| Neg42 | surgery | Neg | 2.27918015 |
| Neg43 | surgery | Neg | 2.26324387 |
| Neg44 | surgery | Neg | 2.04059252 |
| Neg45 | Hospitalization + ER | Neg | 2.33443358 |
| Neg46 | No medical events | Neg | 1.11218971 |
| Neg47 | Meds added/uptitrated | Neg | 3.1378321 |
| Neg48 | No medical events | Neg | 2.39992885 |
| Neg49 | Hospitalization + ER | Neg | 2.68893778 |
| Neg50 | surgery | Neg | 1.46064567 |
| Neg51 | Meds added/uptitrated | Neg | 1.68012273 |
| Neg52 | Hospitalization + ER | Neg | 2.40436659 |
| Neg53 | surgery | Neg | 0.46505868 |
| Neg54 | surgery | Neg | 2.33546424 |
| Neg55 | Hospitalization + ER | Neg | 0.49699324 |
| Neg56 | Hospitalization + ER | Neg | 2.83155036 |
| Neg57 | Hospitalization + ER | Neg | 2.70820596 |
| Neg58 | Hospitalization + ER | Neg | 3.26773235 |
| Neg59 | surgery | Neg | 2.83147448 |
| Neg60 | Meds added/uptitrated | Neg | 1.43954482 |
| Neg61 | Meds added/uptitrated | Neg | 3.4795319 |
| Neg62 | Meds added/uptitrated | Neg | 1.17207638 |
| Neg63 | Hospitalization + ER | Neg | 2.32739626 |
| Neg64 | No medical events | Neg | 3.75399659 |
| Neg65 | Meds added/uptitrated | Neg | 2.89424266 |
| Neg66 | surgery | Neg | 2.24008121 |
| Neg67 | Meds added/uptitrated | Neg | 2.90471194 |
| Neg68 | Meds added/uptitrated | Neg | 2.42020629 |
| Neg69 | Meds added/uptitrated | Neg | 2.3568699 |
| Neg70 | Meds added/uptitrated | Neg | 2.81469896 |
| Neg71 | Hospitalization + ER | Neg | 2.94990095 |
| Neg72 | Meds added/uptitrated | Neg | 3.55253615 |
| Neg73 | No medical events | Neg | 3.72711942 |
| Neg74 | Meds added/uptitrated | Neg | 3.43039587 |
| Neg75 | No medical events | Neg | 3.38659107 |
| Neg76 | Meds added/uptitrated | Neg | 2.07399154 |
| Neg77 | No medical events | Neg | 2.94302927 |
| Neg78 | Meds added/uptitrated | Neg | 3.18886548 |
| Neg79 | Meds added/uptitrated | Neg | 2.96408283 |
| Neg80 | Meds added/uptitrated | Neg | 2.41352529 |
| Neg81 | No medical events | Neg | 2.49122506 |
| Neg82 | Hospitalization + ER | Neg | 1.18700853 |
| Neg83 | Meds added/uptitrated | Neg | 3.47455541 |
| Neg84 | surgery | Neg | 2.38537975 |
| Neg85 | No medical events | Neg | 1.12184465 |
| Neg93 | No medical events | Neg | 2.28261125 |
| Noro38 | Hospitalization + ER | Noro | 2.28964 |
| Noro47 | No medical events | Noro | 1.68985241 |
| Noro48 | Hospitalization + ER | Noro | 2.95394485 |
| Noro54 | No medical events | Noro | 2.34561222 |
| Noro57 | No medical events | Noro | 2.24088558 |

| B | .y. | group1 | group2 | n1 | n2 | statistic | df | p |
| --- | --- | --- | --- | --- | --- | --- | --- | --- |
| 1 | shannon | No medical events | Meds added/uptitrated | 25 | 24 | 0.31551654 | 46.7961242 | 0.754 |
| 2 | shannon | No medical events | Hospitalization + ER | 25 | 19 | 1.77697204 | 41.6678041 | 0.083 |
| 3 | shannon | No medical events | surgery | 25 | 15 | 2.79040977 | 30.4282891 | 0.009 |
| 4 | shannon | Meds added/uptitrated | Hospitalization + ER | 24 | 19 | 1.53253564 | 40.0252554 | 0.133 |
| 5 | shannon | Meds added/uptitrated | surgery | 24 | 15 | 2.60594 | 28.1737471 | 0.015 |
| 6 | shannon | Hospitalization + ER | surgery | 19 | 15 | 1.28650371 | 27.6337609 | 0.209 |

| C | .y. | group1 | group2 | n1 | n2 | statistic | df | p |
| --- | --- | --- | --- | --- | --- | --- | --- | --- |
| 1 | shannon | No medical events | Meds added/uptitrated | 11 | 21 | -0.1866203 | 16.4992426 | 0.854 |
| 2 | shannon | No medical events | Hospitalization + ER | 11 | 10 | 0.52944759 | 18.8644742 | 0.603 |
| 3 | shannon | No medical events | surgery | 11 | 12 | 1.41842642 | 19.3800302 | 0.172 |
| 4 | shannon | Meds added/uptitrated | Hospitalization + ER | 21 | 10 | 0.88375529 | 16.7567076 | 0.389 |
| 5 | shannon | Meds added/uptitrated | surgery | 21 | 12 | 2.09181036 | 22.0735131 | 0.048 |
| 6 | shannon | Hospitalization + ER | surgery | 10 | 12 | 0.9384721 | 19.166772 | 0.36 |

| D | .y. | group1 | group2 | n1 | n2 | statistic | df | p |
| --- | --- | --- | --- | --- | --- | --- | --- | --- |
| 1 | shannon | No medical events | Meds added/uptitrated | 14 | 6 | 0.85423754 | 9.40046552 | 0.414 |
| 2 | shannon | No medical events | Hospitalization + ER | 14 | 10 | 1.59935945 | 20.0634172 | 0.125 |
| 3 | shannon | No medical events | surgery | 14 | 5 | 1.29197129 | 5.65807471 | 0.247 |
| 4 | shannon | Meds added/uptitrated | Hospitalization + ER | 6 | 10 | 0.43768272 | 10.152981 | 0.671 |
| 5 | shannon | Meds added/uptitrated | surgery | 6 | 5 | 0.58915955 | 7.35584178 | 0.573 |
| 6 | shannon | Hospitalization + ER | surgery | 10 | 5 | 0.30606603 | 6.09624822 | 0.77 |
